# Scalable Fabrication of Lightweight, Thermally Insulating and Ablation-Resistant SiC/SiOC Hybrid Aerogel Composites Reinforced with High-Porosity Needle-Punched Quartz Fiber Felts

**DOI:** 10.3390/polym18212694

**Published:** 2026-10-30

**Authors:** Furui Sun, Yang Yu, Bing Wang, Yi Liu, Liuchun Zheng

**Affiliations:** 1 School of Electronics and Information Engineering, Tiangong University, Tianjin 300387, China; 2 State Key Laboratory of Separation Membranes and Membrane Processes, School of Textile Science and Engineering, Tiangong University, Tianjin 300387, China

**Keywords:** SiC/SiOC aerogel, high-porosity needle-punched quartz fiber felts, thermal protection, lightweight, thermal insulation, ablation resistance

## Abstract

High-speed flight demands thermal protection materials combining lightweightness, high strength, efficient thermal insulation, and long-term oxidative ablation resistance. Herein, a ternary hybrid preceramic polymer system consisting of liquid polycarbosilane, polymethylhydrosiloxane, and 1,3,5,7-tetramethyl-1,3,5,7-tetravinylcyclotetrasiloxane was designed through precise control of molecular structure and polymerization kinetics. The composite precursor sol was infiltrated into a high-porosity needle-punched quartz fiber felt via the resin transfer molding method, yielding an integrated thermal protection/insulation material with a bulk density below 0.40 g cm^−3^. The resulting composite exhibited a thermal conductivity as low as 0.042 W m^−1^ K^−1^ at room temperature and 0.082 W m^−1^ K^−1^ at 1000 °C, along with a compressive strength of 7.97 MPa at 10% strain, demonstrating outstanding mechanical performance. In ground-based arc-jet ablation tests (500 MJ m^−2^, 1500 s, ~1400 °C surface), LHRC-05 showed a back-face temperature rise of only 160.5 °C across a 25 mm thick specimen, with a linear ablation rate as low as 0.53 μm s^−1^, accompanied by a mass ablation rate of 0.021 g s^−1^ (near-zero volumetric recession). These results demonstrated that the fiber-reinforced SiC/SiOC aerogel composite integrated low density, high mechanical strength with efficient thermal insulation and excellent ablation resistance, making it a promising candidate for thermal protection systems in aerospace applications.

## 1. Introduction

High Mach number vehicles face extreme coupled thermal, mechanical, and oxidative loads during atmospheric re-entry and long-duration cruise. The aerodynamic heating drives the stagnation temperature typically beyond 1000 °C, and for re-entry trajectories, the surface heat flux can reach several megawatts per square meter, with peak surface temperatures approaching 2000 °C [1]. In the heat flux intensive regions, thermal protection materials must withstand a sustained service at surface temperatures up to ~1400 °C while reconciling several conflicting requirements, namely low density, high load-bearing capacity, efficient thermal insulation, and durable ablation resistance. A failure in any of these properties would directly compromise the structural integrity and flight safety of the vehicles. Although ablative thermal protection systems based on phenolic composites have been applied in re-entry vehicles [2], the rapid oxidation of the carbon-rich skeleton under oxidizing ablation conditions and the progressive degradation of thermal insulation at elevated temperatures remain severe obstacles to long-term service [3]. The development of lightweight thermal protection materials that are capable of long-duration thermal insulation and ablation resistance in the ~1400 °C service regime is therefore of critical importance for next-generation aerospace vehicles.

Aerogels, which combine ultra-low density, high specific surface area, high porosity, and low thermal conductivity, are regarded as promising candidates for thermal insulation purposes [4]. Nevertheless, the compositional constraints of conventional systems limit their high-temperature applicability. Phenolic-based aerogel composites, although widely employed as lightweight ablative insulators, suffer from severe oxidation of carbon-rich skeletons under oxidizing ablation conditions [3]. Silica aerogels, by contrast, exhibit good oxidation resistance but undergo pronounced sintering and densification above 800 °C. The collapse of the nanoporous network in silica aerogels degrades their insulating performance. In this context, polymer-derived ceramic (PDC) aerogels have attracted increasing attention, since their ceramic backbones integrate ultra-low density, tailorable pore structure, high-temperature stability, and low thermal conductivity [4]. Representative examples include SiC/C aerogels derived from hydrosilylation-crosslinked polycarbosilane (PCS) precursors, which retain porosities above 90 vol% and excellent thermal stability [5]. The ceramic nanofibrous aerogels prepared by freeze drying exhibit temperature-invariant superelasticity [6]. However, most PDC aerogels are still produced via supercritical drying [5,7] or freeze-drying [6,8] methods, which require specialized equipment and high energy consumption with complicated processing that limit their scalability for engineering applications. Although reaction-induced phase separation enabled the ambient pressure drying of PCS-derived SiC monoliths [9], and mechanically strong carbon aerogels were realized under ambient conditions [10], the cost-effective ambient pressure synthesis of high-strength ceramic aerogels remains a formidable challenge. In addition, although hydrosilylation of preceramic polymers was previously exploited to prepare polysiloxane and polycarbosilane aerogels [7], those materials were limited to monolithic, supercritical or freeze-dried forms without a demonstrated route to fiber-reinforced composites. The present work advances this chemistry by introducing a ternary PCS/PHMS/V4 system in which PHMS acts not only as a crosslinker but also as an Si–O source that enriches the SiOC fraction by enabling ambient pressure drying and by delivering quantified mechanical and ablation benefits in a fiber-reinforced form (compressive strength 7.97 MPa; linear ablation rate 0.53 μm s^−1^).

Despite the attractive properties of monolithic aerogels, their practical application is restricted by the intrinsic brittleness of the ceramic network and the weak interfacial bonding between the aerogel matrix and reinforcing skeleton. Fiber felt-reinforced aerogel composites overcome the above limitations by integrating the mechanical robustness and damage tolerance of continuous fiber felts with the thermal insulative aerogel matrix [3,11]. For instance, needle-punched quartz fiber felt-reinforced SiOC phenolic aerogel composites exhibit bulk densities of 0.30–0.35 g cm^−3^ along with a maximum compressive strength of 4.20 MPa [3]; carbon fiber felt-reinforced C/SiOC aerogel composites combine a tunable density of 0.25–0.40 g cm^−3^ with compressive strengths of up to 10.37 MPa [11], and flexible carbon fiber felt-reinforced phenolic impregnated carbon ablators can be folded more than 500 times without fracture [2]. ZrO_2_ fibrofelt/ZrO_2_–SiO_2_ aerogel composites enabled large-scale fabrication with ultra-low thermal conductivity [12]. Moreover, functionally gradient fiber felt architectures, such as gradient quartz fiber-needled felts, have proven effective in realizing integrated anti-ablation and thermal insulation performance [13]. Nevertheless, most reported fiber-reinforced aerogel composites rely on oxide- or carbon-based matrices, whose high-temperature stability and oxidation resistance remain inadequate for long-duration service in oxidizing environments. Although the ZrO_2_ fibrofelt/ZrO_2_–SiO_2_ composites [12] achieved large-scale fabrication and the gradient quartz fiber felt composites [13] realized integrated anti-ablation and insulation, both depend on oxide-dominated matrices: the ZrO_2_-based system possesses a relatively high intrinsic thermal conductivity and limited ablation resistance at ~1400 °C, whereas the gradient felt system in [13] relies on a SiO_2_-based ceramic/aerogel matrix whose protection is governed mainly by the sacrificial felt architecture rather than by a self-healing silicon carbide-derived ceramic phase. A systematic and scalable molding route that simultaneously reconciles lightweightness, high strength, efficient insulation, and ablation resistance in a silicon-based (SiC/SiOC) hybrid matrix therefore remains lacking—the gap addressed by the present work. In this study, a high-porosity needle-punched quartz fiber felt is employed as the reinforcing preform, which is the high-porosity needle-punched quartz fiber felt architecture referred to throughout the manuscript.

In this work, the PHMS content within the PCS/PHMS/V4 ternary precursor system was modulated to enable controlled tailoring of the micro/nanoscale pore structure of SiC/SiOC hybrid aerogels and further refine the three-dimensional interpenetrating network. Ambient pressure drying was established as a viable route for the scalable fabrication of lightweight SiC/SiOC hybrid aerogels; within the present study the largest panel fabricated measured 330 × 330 × 40 mm with a batch-to-batch density variation of 5%, and a full engineering-scale demonstration remains the subject of ongoing work. The optimized composite (LHRC-05) achieved a compressive strength of 7.97 MPa at 10% strain, representing a 319% enhancement relative to the pure PCS-derived SiC matrix (LHRC-0), while the high-porosity needle-punched quartz fiber felt-reinforced sample delivered lightweightness, high mechanical strength, efficient thermal insulation, and excellent ablation resistance. This work provides a technical solution for the scalable fabrication of integrated thermal protection materials for combating extreme environments in the aerospace industry and other heat-shielding sectors.

## 2. Materials and Methods

### 2.1. Materials

Liquid polycarbosilane (LPCS, M_n_: 1100–1300 g/mol) was purchased from the Institute of Chemistry, Chinese Academy of Sciences (Beijing, China). Polymethylhydrosiloxane (PHMS, active hydrogen mass fraction: 1.5–1.6 wt%) and Tetravinyltetramethylcyclotetrasiloxane (V4, purity ≥ 99 wt%, vinyl mass fraction: 32%) were provided by Shanghai Macklin Biochemical Co., Ltd. (Shanghai, China). Karstedt’s platinum catalyst (Pt-vinylsiloxane complex, platinum mass fraction: 3000 ppm) was supplied by Shanghai Aladdin Biochemical Technology Co., Ltd. (Shanghai, China). N-hexane (purity ≥ 97 wt%) was obtained from Sinopharm Chemical Reagent Co., Ltd. (Shanghai, China). A high-porosity needle-punched quartz fiber felt (bulk density 0.2 g cm^−3^) was provided by Tianniao High Technology Co., Ltd. (Wuxi, China). The felt was fabricated by cross-laminating staple quartz fiber webs and quartz fabric layers. Its characteristic parameters are listed as follows: total thickness of 40 mm, areal densities of 136 g m^−2^ and 80 g m^−2^ for the fabric layers, mean fiber diameter ranging from 5 to 10 μm. The inter-fiber porosity of the felt was determined from the apparent bulk density (ρ_b_) and the true density of quartz fibers (=2.20 g cm^−3^) as ε = (1 − ρ_b_/ρ_f_) × 100%. For the felt panel employed here (mass m = 875.6 g, 330 × 330 × 40 mm), ρ_b_ = m/V ≈ 0.201 g cm^−3^, yielding a total inter-fiber porosity of ≈90.9%. This density-based value represents the total inter-fiber porosity of the felt: the inter-fiber voids are predominantly interconnected open pores, together with a small proportion of isolated closed voids enclosed by overlapping staple fibers, both of which are counted within this total porosity. High-purity argon (Ar ≥ 99.999%) was supplied by Beijing Praxair Industrial Gas Co., Ltd. (Beijing, China).

### 2.2. Fabrication of PCS/PHMS/V4-Derived SiC/SiOC Aerogels

Liquid PCS and PHMS were dissolved in n-hexane at predetermined mass ratios, and the mixture was magnetically stirred for 30 min to yield a homogeneous optically transparent precursor solution. The crosslinker V4 was added at 1.5 times the total mass of PCS and PHMS (m(V4) = 1.5 × [m(PCS) + m(PHMS)]), supplying an excess of vinyl groups to drive the hydrosilylation crosslinking. Four formulations, which were designated as SP025, SP050, SP075, and SP1, were prepared with PCS/PHMS mass ratios of 1:0.25, 1:0.5, 1:0.75, and 1:1, respectively. A platinum catalyst solution (0.1 wt%, relative to the total mass of PCS, PHMS and V4) was subsequently introduced, and the mixture was stirred vigorously for 15 min to ensure homogeneous catalyst dispersion. The precursor solution was transferred into a 20 mL high-temperature-resistant sealed vessel. The vessel was subject to a programmed thermal treatment in a forced-air oven: (i) heated at 1 °C/min to 80 °C and held isothermally for 4 h; (ii) further heated at 1 °C/min to 120 °C and held isothermally for 8 h; and (iii) heated at 3 °C/min to 180 °C and held isothermally for 12 h to ensure complete crosslinking and gel formation. A K-type thermocouple was mounted on the vessel exterior to monitor the temperature profile throughout gelation process. Upon natural cooling to ambient temperature, the wet gel was subsequently dried as per a two-stage drying protocol: at room temperature for 24 h; 60 °C at 8 h and followed by 120 °C at 6 h, yielding PCS/PHMS-based aerogels. The reference PCS aerogel which was designated as SP0 was prepared by following the same procedure. A functional group material balance for the PCS/PHMS/V4 formulations was computed from the reactive group contents of the raw materials: PHMS with an active hydrogen (Si–H) mass fraction of 1.5–1.6 wt%, V4 with a vinyl (Si–CH=CH_2_) mass fraction of 32 wt%, and PCS with a vinyl end group content (0.2–1 wt%) and a Si–H content (0.1–1.5 wt%) as specified by the supplier. The balance shows that, at the fixed V4 loading of 1.5 times the total PCS + PHMS mass, vinyl groups remain in excess of Si–H across the SP025–SP1 series (Si–H: vinyl molar ratio ≈ 0.53–0.66 for a representative PCS composition, increasing with PHMS loading); Si–H is therefore the limiting reagent, and the hydrosilylation crosslinking proceeds toward completion with excess vinyl retained. The present formulation thus avoids assuming a constant Si–H: vinyl ratio and underpins the proposed hydrosilylation crosslinking mechanism.

The dried precursor aerogels were placed in alumina crucibles and transferred to a tube furnace for high-temperature conversion from organic skeletons to ceramic skeletons via precursor infiltration and pyrolysis (PIP) [14,15]. Pyrolysis was conducted under a purging argon atmosphere to prevent high-temperature oxidation. The staged heating profile was set as follows: room temperature to 300 °C at 1 °C/min, 300–900 °C at 3 °C/min, and 900–1400 °C at 5 °C/min. After pyrolysis treatment, samples were cooled to ambient temperature under argon to obtain ceramic aerogels with different starting PHMS content. The samples were then labeled as SPA0, SPA025, SPA050, SPA075, and SPA1, corresponding to the precursor mass ratios of the organic aerogels.

### 2.3. Preparation of Aerogels with Integrated Ablation Resistance and Thermal Insulation

The high-porosity needle-punched quartz fiber felt was placed into a metallic molding die. A vacuum system was connected for 1 h to evacuate residual air within the pores of the fiber network. The above-formulated ternary hybrid precursor sol (Section 2.2) was injected into the die via resin transfer molding (RTM) to achieve full impregnation of the fiber reinforcement and inter-fiber voids, a process whose impregnation efficiency is governed by the anisotropic permeability and void network of the needle-punched felt, as analyzed for RTM in porous fiber preforms [16,17]. The sealed mold was transferred into a programmable oven for in situ sol–gel transition: (1) the system was heated to 80 °C at 1 °C/min and held for 4 h; (2) it was subsequently elevated to 120 °C at 1 °C/min and held for 8 h; (3) it was finally heated to 180 °C at 3 °C/min and maintained for 12 h to form the three-dimensional organic/inorganic hybrid network. Consequently, a lightweight, heat-insulating and reinforced composite (LHRC) aerogel was obtained. The as-prepared LHRC possessed a bulk density of approximately 0.4 g/cm^3^ and a porosity ≥ 75%. The aerogel composite achieved integrated performance, including lightweightness, ablation resistance, efficient thermal insulation, and load-bearing capability. After pyrolysis at 1400 °C, the SP-series organic aerogels were redesignated as SPA-series ceramic aerogels. The optimized SP050 was then infiltrated into the quartz felt to yield LHRC-05, while SP0 served as the reference monolithic (LHRC-0).

### 2.4. Characterizations

The bulk density of samples was measured by dividing mass by volume as per GB/T 1463-2005 [18]. Field emission scanning electron microscopy (FE-SEM, ZEISS GeminiSEM 360, ZEISS, Oberkochen, Germany) was used to observe the microstructure of hybrid aerogels and the cross-sectional morphology of composites. Specimens were gold-sputtered prior to testing to enhance surface electrical conductivity. Pore structures were characterized via an automatic mercury intrusion porosimeter (Micromeritics AutoPore V 9620, Micromeritics Instrument Corporation, Norcross, GA, USA) over a pressure range of 0–228 MPa. Fourier transform infrared spectroscopy (FT-IR, Nicolet iS50, Thermo Fisher Scientific, Waltham, MA, USA) was used to analyze the functional groups and chemical bond evolution of hybrid systems across a scanning range of 4000–400 cm^−1^. Thermal conductivity was tested using a thermal constant analyzer (TPS2500S, Hot Disk, Hot Disk AB, Göteborg, Sweden) as per ISO 22007-2:2022 [19]. Three parallel groups were tested to obtain average values, and the standard deviations are reported as error bars. At elevated temperature, the thermal conductivity measurements were conducted in Ar atmosphere. Compressive properties were measured on an electronic universal testing machine (WDW-10, MTS Systems Co., Ltd., Shanghai, China) as per GB/T 1448-2005 [20]. Five specimens were tested per group, and the arithmetic mean values were reported. The ablation tests were conducted using a plasma arc-jet wind tunnel. A stepped heating profile was applied: an initial heat flux of 100 kW m^−2^ for 300 s, followed by a hold at 400 kW m^−2^ for 1200 s, giving a total exposure duration of 1500 s and a cumulative heat load of approximately 500 MJ m^−2^. Detailed parameters of test items with special requirements are shown in Table 1.

## 3. Results and Discussion

### 3.1. Microstructure and Chemical Compositions of PCS/PHMS/V4 Precursor Aerogels

**Figure 1:**
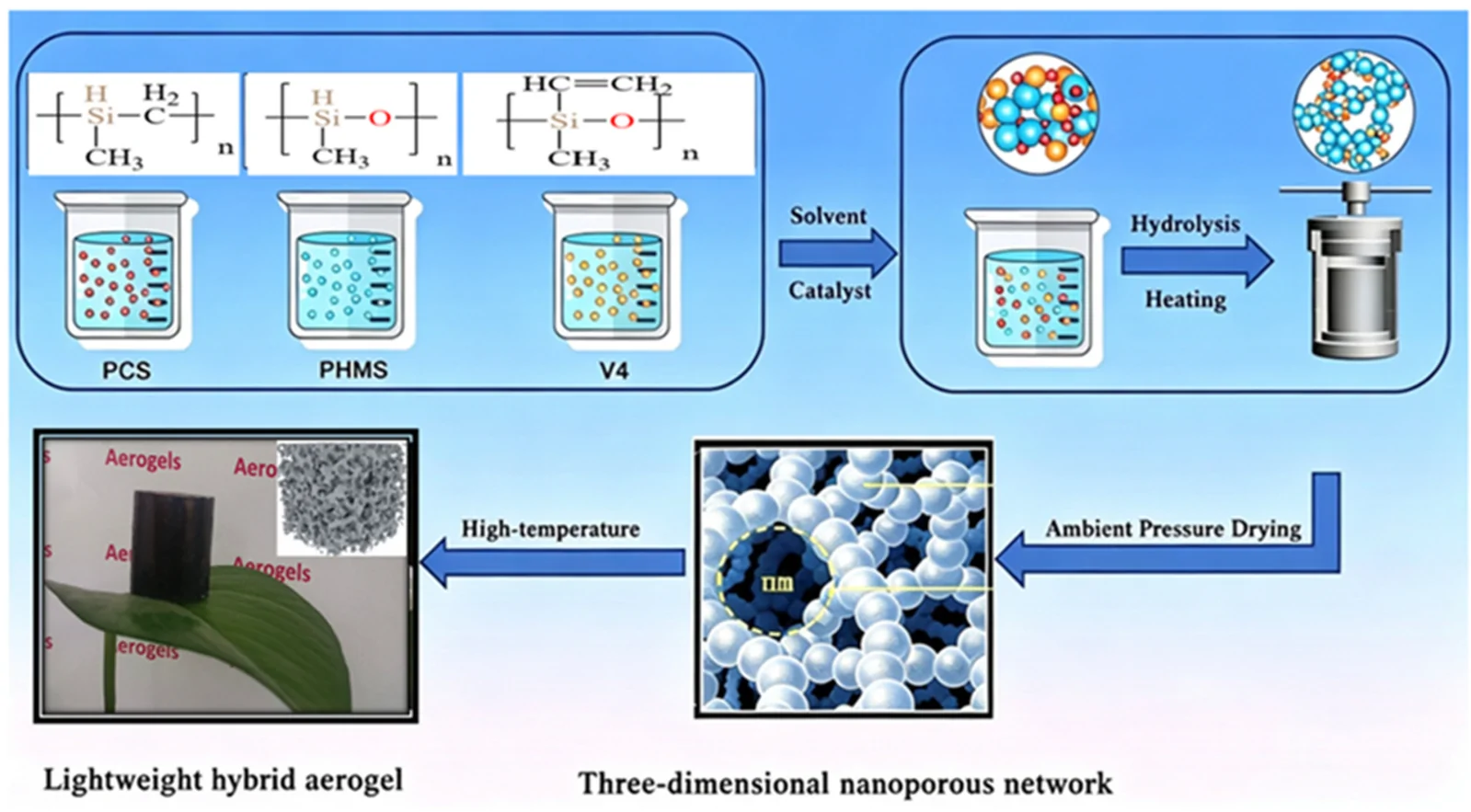
Schematic diagram of preparing SiC/SiOC aerogel composites.

**Figure 2:**
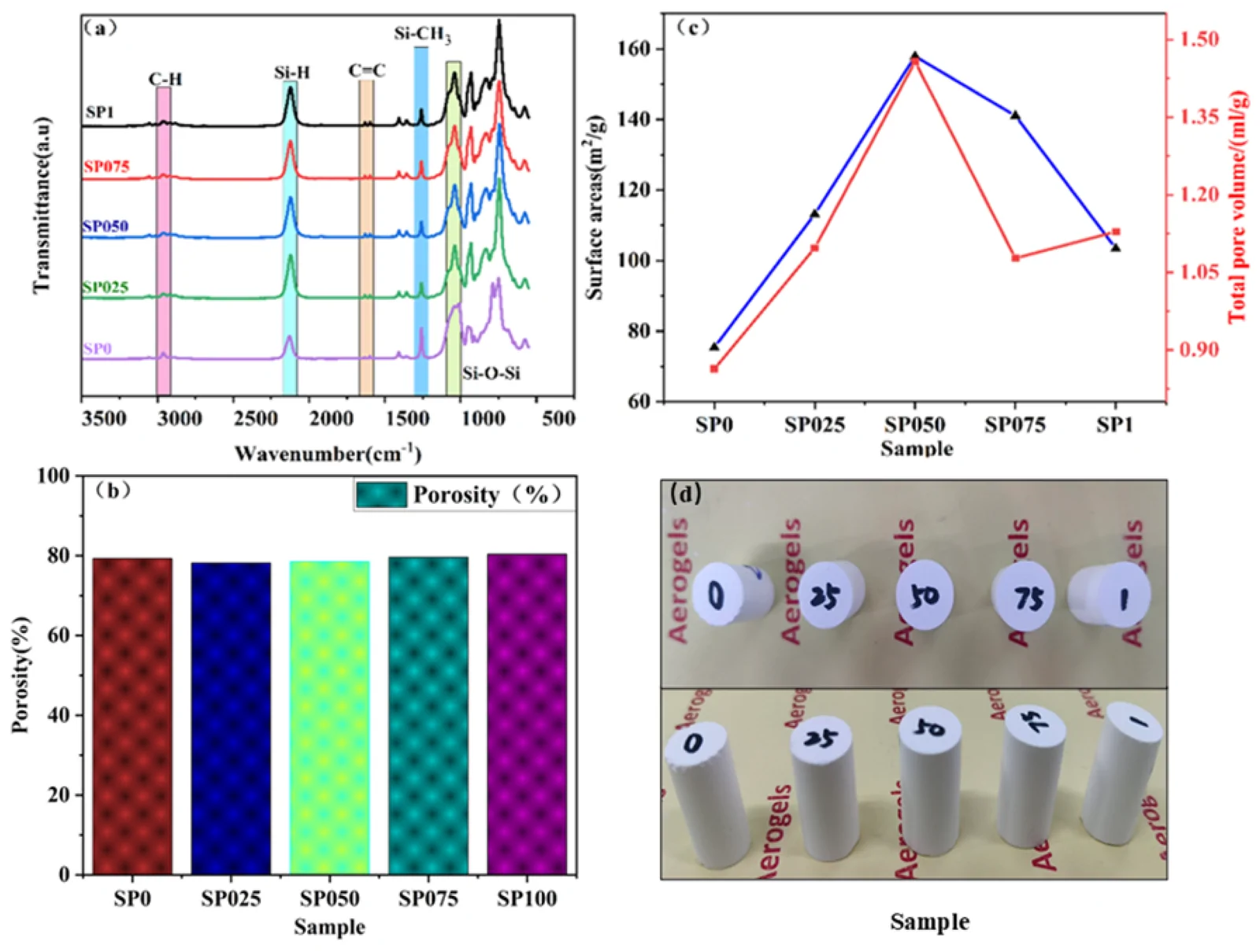
(**a**) FT-IR spectra of aerogel before pyrolysis. (**b**,**c**) BET results and porosity of aerogels. (**d**) Images of the aerogel monoliths prepared using the atmospheric pressure drying process.

A series of PCS/PHMS/V4 hybrid silicon-based aerogels with gradient PHMS loadings were fabricated via a standard sol–gel route that is suitable for scale-up [21,22,23], and the detailed fabrication procedure is presented in Figure 1. During the gelation stage, the Si–H bonds in PHMS side chains underwent hydrosilylation with the C=C double bonds in V4, while the reactive sites on PCS chains simultaneously participated in the formation of a three-dimensional network, enabling uniform molecular-scale hybridization through Si-C covalent bonds. The skeleton flexibility and pore-size distribution of the hybrid aerogels can be tuned by the PHMS loading. FT-IR spectra of PCS/PHMS/V4 hybrid precursor aerogels with different PHMS loadings verified the progression of the hydrosilylation reaction at the molecular scale [24,25], as shown in Figure 2a. The SP0 exhibited a distinct Si–CH=CH_2_ stretching vibration at 1605 cm^−1^, assigned to the vinyl groups in V4 and on PCS side chains. Peaks at 2960 and 1260 cm^−1^ were attributed to C–H stretching and bending of Si–CH_3_, respectively, while the broad band from 1000 to 1120 cm^−1^ corresponded to the asymmetric stretching of Si–O–Si. The vinyl peak at 1605 cm^−1^ was gradually attenuated and essentially disappeared at the optimal PHMS loading (SP050). Concurrently, the C–H stretching of Si–CH_3_ intensified, originating partly from methyl side chains in PHMS and mainly from the methylene C–H of Si–CH_2_–CH– formed by hydrosilylation. The peak of the Si–O–Si band broadened and strengthened with increasing PHMS content, reflecting enhanced crosslinking, higher polymerization degree, and improved network continuity. The results confirmed that the active Si–H bonds on PHMS chains reacted with vinyl groups, constructing a three-dimensional crosslinked network through Si–CH_2_–CH_2_–Si bonds. The strengthened three-dimensional skeleton mitigated pore structure damage caused by capillary forces during ambient pressure drying, suppressing skeleton collapse and volumetric shrinkage and enabling industrial-scale drying of the hybrid aerogels. Optical images of the dried monoliths are shown in Figure 2d.

**Figure 3:**
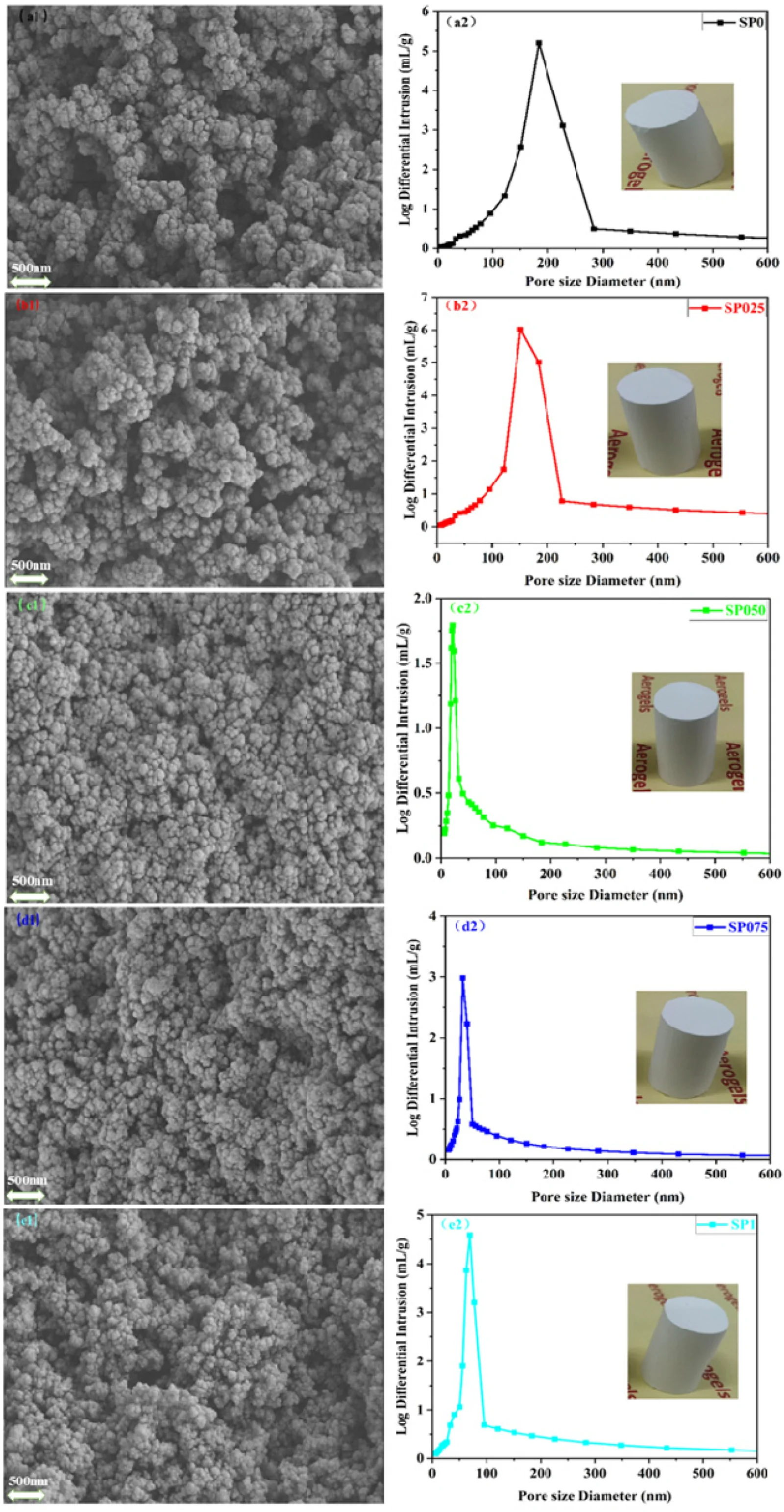
SEM images of aerogels with different PHMS content: (**a1**) SP0, (**b1**) SP025, (**c1**) SP050, (**d1**) SP075, (**e1**) SP1. The pore size distribution of aerogels with different PHMS content: (**a2**) SP0; (**b2**) SP025; (**c2**) SP050; (**d2**) SP075; (**e2**) SP1.

The pore structure of SP-series aerogels was characterized by mercury intrusion porosimetry and BET measurements [26,27]. As shown in Figure 2b,c and Figure 3a2–e2, the specific surface area first increased and then declined with increasing PHMS content, whereas the porosity exhibited no obvious variation. A moderate PHMS addition regulates the agglomeration of secondary particles to construct a multi-scale interpenetrating network, thereby boosting the specific surface area. Mercury intrusion tests revealed that the reference sample fell within the macroporous range (>50 nm), with a broad pore size distribution, large average pore size, and low pore volume. With increasing PHMS content, the average pore size gradually shifted toward a mesoporous range (2–50 nm), accompanied by a narrower distribution. The total pore volume likewise first increased and then decreased. This trend is consistent with the SEM observations: a higher crosslinking density refined primary particles and promoted denser chain packing, yielding smaller more uniformly distributed pores. An excess PHMS addition, however, accelerated crosslinking to trigger localized premature gelation and phase separation, inducing abnormal particle agglomeration and macropore defects that increased the average pore size and reduced both the specific surface area and the total pore volume.

SEM observations of hybrid precursor aerogels with different PHMS loadings revealed characteristic three-dimensional networks stacked by nanoparticles, with pronounced differences in particle size, network connectivity, and pore uniformity. The microstructure of the reference sample is shown in Figure 3(a1). Severe agglomeration of ceramic particles, abundant uneven large voids, and poor skeleton continuity were observed in SP0, demonstrating a loose structure. Compared with SP0, the PHMS-containing samples shown in Figure 3(b1–e1) exhibited progressively refined primary particles, stronger interparticle necks, and markedly improved continuity and uniformity of the three-dimensional network. At the optimal PHMS loading, the average particle size was reduced to the nanoscale, with uniform pore distribution and maximum skeleton integrity. Excess PHMS loading induced abnormal agglomeration of secondary particles, giving rise to macropore defects and compromising the uniformity of the three-dimensional network. PHMS possesses abundant active Si–H bonds that undergo hydrosilylation with V4 and with the unsaturated C=C terminal bonds in PCS, introducing numerous covalent hybrid crosslinking sites at the molecular scale [28,29].

### 3.2. Properties of PCS/PHMS/V4-Derived SiC/SiOC Aerogels

**Figure 4:**
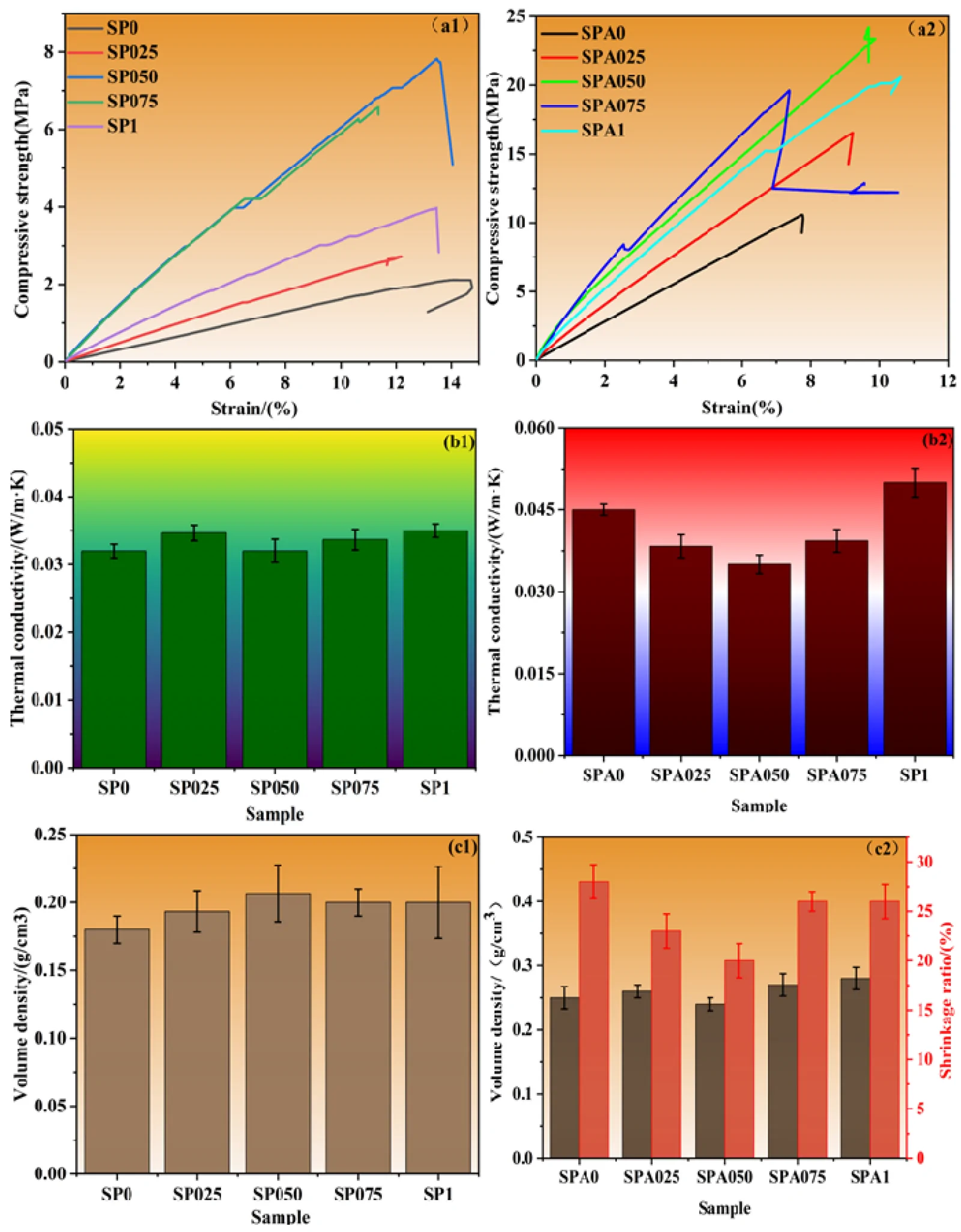
Stress–strain curves of hybrid aerogels with different PHMS contents before and after pyrolysis: (**a1**) SP, (**a2**) SPA. Thermal conductivity of hybrid aerogels with different PHMS contents before and after pyrolysis: (**b1**) SP; (**b2**) SPA. Density and linear shrinkage of hybrid aerogels with different PHMS contents before and after pyrolysis: (**c1**) SP; (**c2**) SPA.

The hybrid precursor aerogels were fabricated by incorporating varying amounts of PHMS into PCS via sol–gel and ambient pressure drying (APD) methods, and the PHMS-mediated regulation of mechanical properties, thermal insulation, and high-temperature stability was investigated [30,31]. The performance data are summarized in Table 2 and Figure 4(a1). For the precursor aerogels, the bulk density, compressive strength, and compressive modulus followed a unimodal trend of first increasing and then decreasing with PHMS loading, with an optimum at a PCS/PHMS mass ratio of 1:0.5. The pure PCS aerogel (SP0) had insufficient crosslinking sites, with severe molecular chain entanglement and phase separation during sol–gel processing; these effects produced a loose skeletal network with a density of 0.18 g cm^−3^, a compressive strength of 2.12 MPa, and a compressive modulus of 20 MPa.

Increasing PHMS loading to 0.5 drastically increased the number of hydrosilylation crosslinking nodes, which homogenized the network structure and suppressed phase separation to enhance skeleton load-bearing capacity. The compressive strength and modulus were enhanced by 269% and 250%, respectively, relative to pure PCS aerogels, with density only marginally increasing to 0.21 g/cm^3^, as illustrated in Figure 4(c1). When the PHMS loading was excessively high, unreacted PHMS molecules remained dispersed within the network as low molecular weight oligomers. Acting as plasticizers, these species weakened the crosslinked network and induced localized phase separation, which in turn created structural defects and led to the deterioration of mechanical performance [32].

The room temperature thermal conductivity of the precursor aerogels fell within a narrow range of 0.032–0.035 W m^−1^ K^−1^ (Figure 4(b1)). Heat transfer within the precursor aerogels was dominated by gaseous conduction; all samples maintained high porosity, and the nanopores effectively suppressed thermal convection. Additionally, the solid skeleton volume fraction remained negligible, and solid state variations induced by PHMS incorporation had a limited influence on thermal conductivity [33,34]. When the PHMS loading was excessively high, accelerated agglomeration of secondary particles gave rise to macropores of non-uniform size, resulting in a higher thermal conductivity for SP1 than the optimally formulated sample. Upon pyrolysis at 1400 °C, the organic precursor aerogels underwent bond cleavage, small molecule elimination, and inorganic phase rearrangement, and they were afterwards converted into multiphase SiC/SiOC ceramic aerogels. Volumetric shrinkage reflected the pyrolysis behavior, and it was a decisive factor governing the final structure and properties of ceramic aerogels. As shown in Figure 4(c2), pure PCS aerogel exhibited a linear shrinkage as high as 28% after pyrolysis, whereas PHMS addition markedly suppressed pyrolytic shrinkage, reducing it to 20% at a PCS/PHMS mass ratio of 1:0.5, reflecting superior anti-shrinkage performance. This behavior is ascribed to the formation of a uniform crosslinked network that synchronizes bond cleavage, small molecule release, and structural rearrangement during pyrolysis, thereby avoiding the localized excessive shrinkage and stress concentration caused by non-uniform crosslinking in pure PCS.

The shrinkage suppression afforded by PHMS addition was related to the intrinsically higher bond energy of Si–O (452 kJ mol^−1^) relative to Si–C (318 kJ mol^−1^). The SiOC-rich inorganic skeleton that formed at the early stage of pyrolysis provided rigid support during the decomposition of organic components and the release of small molecules (H_2_ and CH_4_), effectively preventing skeleton collapse and densification. When the PHMS loading exceeded 0.5, the pyrolytic shrinkage rebounded. Excess PHMS induced phase separation of the precursor network, and the structural evolution of Si–O-rich and Si–C-rich phases during pyrolysis exacerbated stress mismatch at the phase interfaces and amplified volumetric change.

After pyrolysis at 1400 °C, the mechanical properties of samples increased markedly relative to the precursor aerogels, owing to the intrinsic strength and modulus gain accompanying the conversion of organic skeletons into inorganic ceramic frameworks. Table 3 and Figure 4(a2) showed that the PHMS-modified multiphase aerogels delivered far superior mechanical performance than that of pure SiC aerogel (SPA0). Specifically, SPA050 achieved a compressive strength of 24.20 MPa, 128.7% higher than SPA0, while SPA075 exhibited a compressive modulus of 360 MPa, 157.1% higher than SPA0, demonstrating effective modulation of the mechanical properties.

The variation in compressive strength is correlated with microstructural homogeneity and defect density. At a PCS/PHMS mass ratio of 1:0.5, the crosslinked precursor network showed optimal uniformity; the pyrolyzed ceramic skeleton contained the fewest defects and well-formed sintering necks between particles, as confirmed by SEM observations, facilitating effective stress transfer within the framework and inhibiting brittle fracture. Heterogeneous interfaces between SiOC-rich and SiC-rich domains within the multiphase skeleton may contribute to fracture energy dissipation and thereby to fracture strength. This crack deflection interpretation is proposed here as a tentative mechanism and is not asserted on the basis of [35,36,37], which are cited only as general background on toughening and do not by themselves constitute direct evidence of crack bridging in the present SiC/SiOC system; phase-specific microstructural confirmation remains beyond the scope of this study. At excessive PHMS loadings, phase separation and carbothermal reduction-derived defects acted as fracture initiation sites, driving sustained strength degradation. Notably, the compressive modulus peaked at a PHMS-to-PCS ratio of 0.75. This trend was rationalized by the intrinsic stiffness contrast between the two constituent phases: increasing the PHMS loading progressively enriched the SiOC fraction within the ceramic skeleton and thereby raised the bulk stiffness. Beyond the optimum, however, excess PHMS perturbed the colloidal assembly during gelation. The secondary clusters coalesced into oversized heterogeneous aggregates rather than remaining uniformly dispersed. These coarsened aggregates broadened the pore size distribution and introduced irregular macropore defects into the otherwise homogeneous three-dimensional network. Under compression, such defects acted as preferred sites for stress concentration and crack initiation. As a result, the skeleton fractured at a noticeably lower strain, and the compressive strength demonstrated a downward trend. At a PCS/PHMS ratio of 1:1, the network non-uniformity became severe enough that the load-bearing paths were substantially interrupted, dropping the modulus back to a level comparable to that of pure SiC aerogel. Across the explored composition window, the optimized sample achieves superior compressive strength relative to conventional SiC ceramic aerogels under low-density conditions, which corroborates the effectiveness of the hydrosilylation-based compounding route.

After pyrolysis, the room temperature thermal conductivity of PHMS-modified multiphase aerogels decreased markedly relative to the pure SiC counterpart, falling from 0.045 to a minimum of 0.035 W m^−1^ K^−1^ at a PCS/PHMS mass ratio of 1:0.5 (SPA050) (Figure 4(b2)). This reduction was rationalized by the synergistic tailoring of pore architecture across the three heat transfer channels governing insulation, namely gaseous conduction, infrared radiative transfer, and solid conduction. With respect to the gaseous channel, the pore size distribution and its evolution upon pyrolysis are decisive. The pure SiC aerogel underwent pronounced linear shrinkage during pyrolysis, which densified the skeleton and coarsened pore network, shifting the pore size distribution toward larger mesopores and macropores. Under such conditions, the pores ceased to constrain the mean free path of air molecules (~70 nm at ambient pressure), and the gas phase conduction approached that of stationary air (~0.026 W m^−1^ K^−1^). In contrast, SPA050 displayed the lowest pyrolytic shrinkage among the studied samples, preserving a high fraction of pores below air molecular mean free path. The retained narrowly distributed nanopores geometrically constrained gas molecule collisions and suppressed gaseous conduction. Concerning the radiative channel, the nanoscale skeleton architecture of SPA050 constituted a strongly scattering medium for mid- and far-infrared thermal radiation. The uniformly distributed nanosized ligaments and interparticle necks generated numerous refractive index discontinuities that scattered photons along their propagation path, thereby attenuating radiative heat transfer. Conversely, the coarsened skeleton and enlarged pores of pure SiC aerogel acted as a semi-transparent medium with less scattering centers, allowing IR radiation to penetrate more deeply and elevate radiative contribution. For the solid channel, the SiC/SiOC skeleton of SPA050 contained abundant heterogeneous phase boundaries between SiC-rich and SiOC-rich domains within the ceramic skeleton. These internal interfaces are proposed to act as phonon-scattering centers that shorten the phonon mean free path within solid ligaments and depress intrinsic solid phase thermal conductivity; this interpretation is consistent with the observed thermal conductivity reduction but has not been verified by phase-resolved spectroscopy and is therefore presented as a proposed mechanism. In pure SiC aerogel, by contrast, the coarsened crystalline network offered fewer such scattering interfaces, while the reduced porosity and thicker ligaments provided more connected conduction paths, both of which raised solid conduction. The combined suppression of the three heat transfer channels accounted for the ~22% reduction in room temperature thermal conductivity from 0.045 to 0.035 W m^−1^ K^−1^.

Beyond the optimum, the thermal conductivity reversed its downward trend, rising progressively to 0.050 W m^−1^ K^−1^ at a 1:1 mass ratio, even exceeding that of pure SiC aerogel. This rebound stemmed from the coarsening of pore architecture at excess PHMS loadings. Intensified pyrolytic shrinkage lowered the porosity and thickened solid ligaments, which reduced phonon-scattering interfaces and lengthened connected solid paths, thus raising solid conduction. Meanwhile, the oversized secondary aggregates broadened the pore size distribution toward mesopores above the air molecular mean free path, recovering gaseous conduction, and the coarsened skeleton diminished infrared scattering centers, thereby enhancing radiative heat transfer. As a result, the thermal conductivity of samples showed an upward trend after surpassing the optimal formulation.

**Figure 5:**
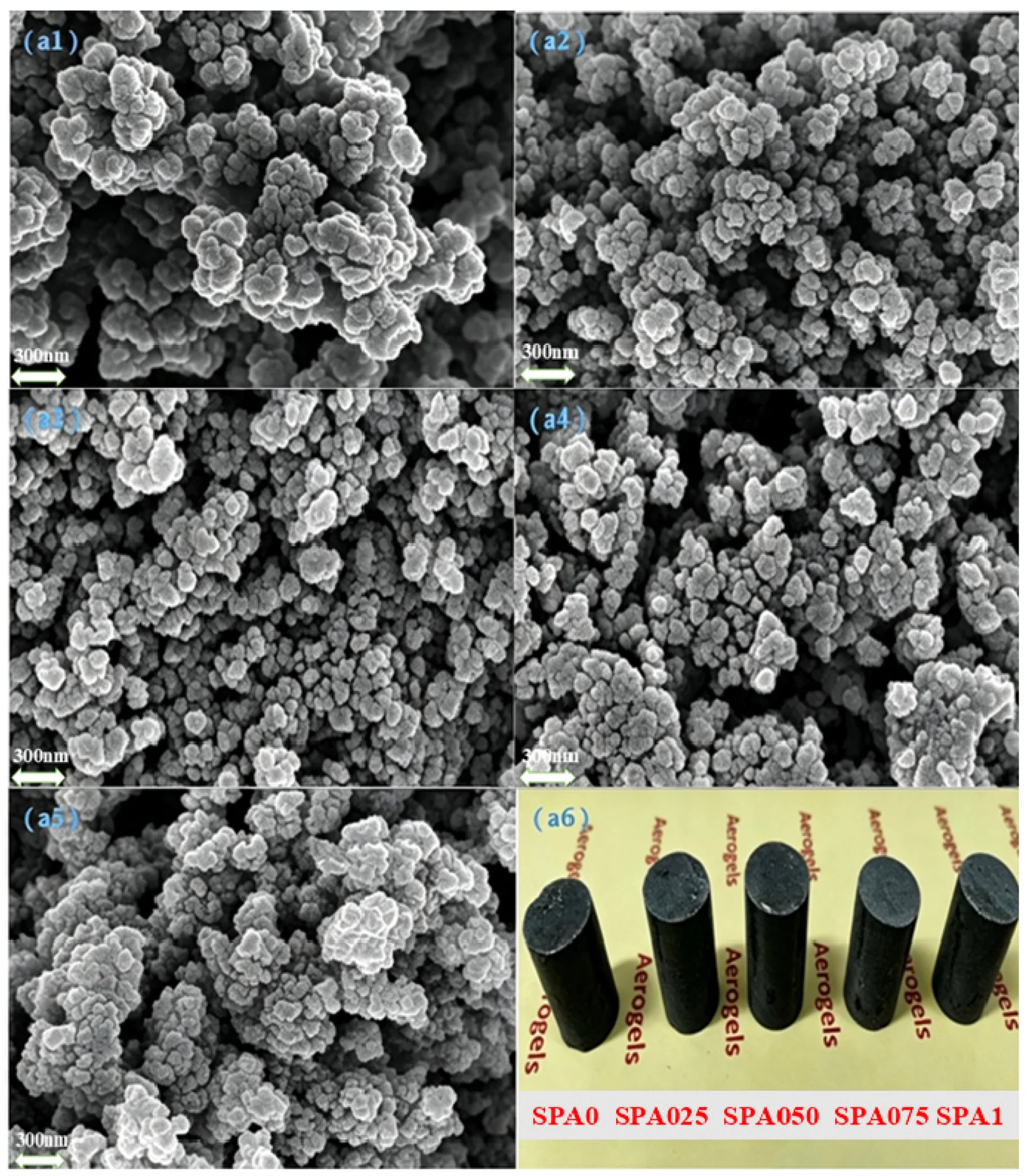
SEM images of aerogels prepared with different ratios of PHMS after pyrolysis. (**a1**) SPA0; (**a2**) SPA025; (**a3**) SPA050; (**a4**) SPA075; (**a5**) SPA1. (**a6**) Photograph of aerogels after high-temperature pyrolysis.

Consistent with the mercury intrusion results, the pyrolyzed samples exhibited three-dimensional porous skeletons constructed by stacked ceramic nanoparticles with interconnected nanopores, as presented in Figure 5(a1–a5). SPA0 displayed severe particle agglomeration, abundant oversized pores, and weak interparticle junctions originating from uneven PCS crosslinking and inconsistent pyrolytic shrinkage, directly accounting for its poor mechanical performance and high thermal conductivity. A moderate PHMS addition (SPA050) yielded uniform ceramic particle sizes, denser stacking, well-developed interparticle neck connections, which markedly improved skeleton continuity, and near-elimination of oversized structural defects. This homogeneous microstructure enabled efficient and uniform stress transfer and provided abundant uniform nanoscale pores, representing the structural origin of synergistically optimized performance. Excess PHMS (SPA1) aggravated particle agglomeration and introduced distinct phase-separated regions, in which partial domains underwent excessive sintering to form particle clusters while the other regions contained oversized pores, thereby disrupting the porous structure and degrading both mechanical and thermal insulation performance. Macroscopically, all samples retained intact monolithic morphology without cracking after pyrolysis, confirming that the skeletal architecture obtained under the optimal additive ratio accommodated the skeletal shrinkage during pyrolysis and that the present route was scalable for bulk sample preparation.

### 3.3. Reinforcement Mechanism of Fiber-Containing SiC/SiOC Aerogel Composites

**Figure 6:**
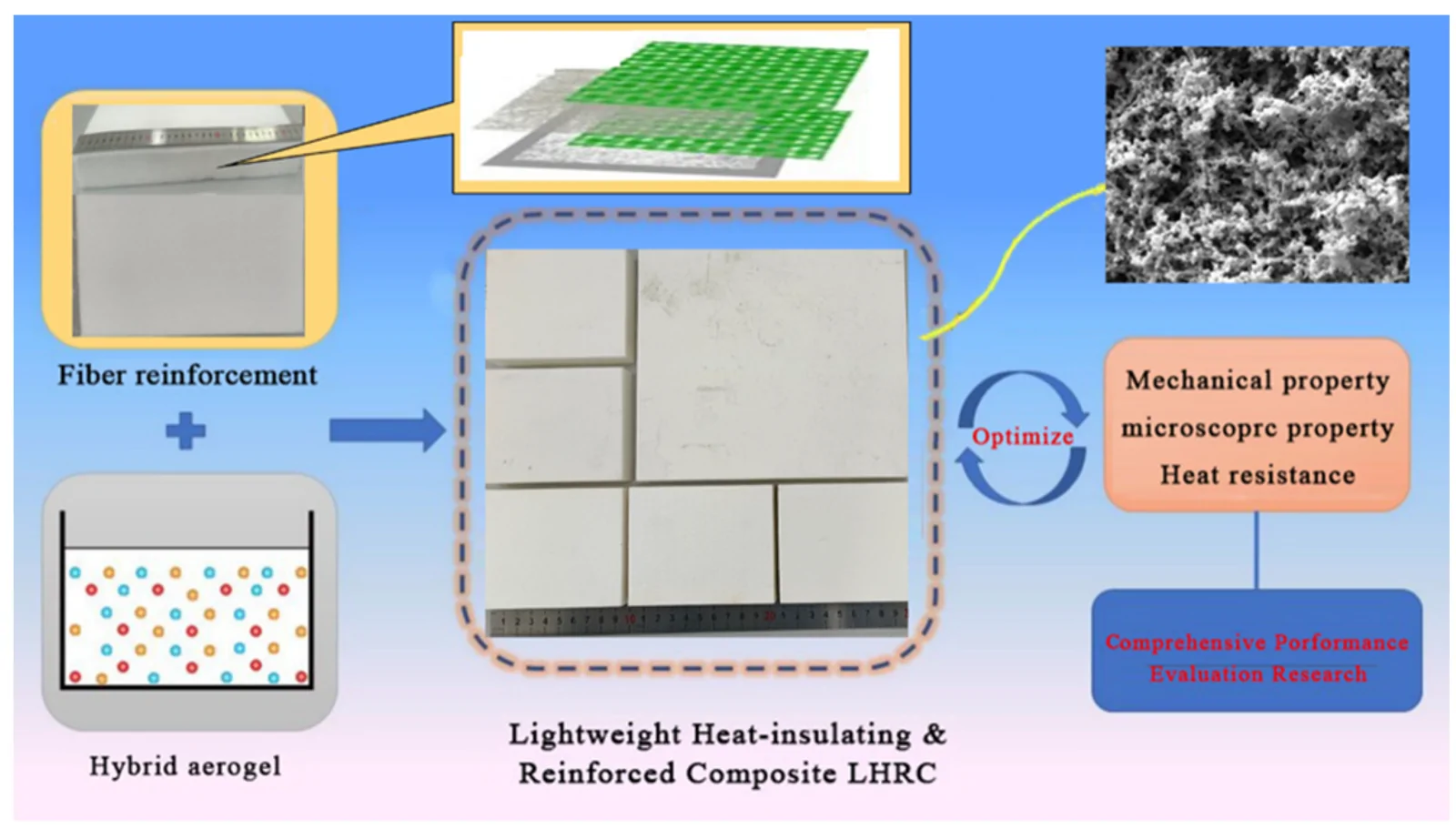
Schematic of the preparation of the high-porosity needle-punched quartz fiber felt-reinforced SiC/SiOC aerogel composites, illustrating the felt architecture, RTM infiltration, in situ gelation, and pyrolysis steps.

Building on the optimized aerogel matrix, this section examined how the high-porosity needle-punched quartz fiber felt reinforced the SiC/SiOC skeleton and how the PHMS-derived chemistry modified the fiber–matrix interface. The optimized aerogel with a PCS/PHMS mass ratio of 1:0.5 served as the resin matrix. A homogeneous sol was formed through platinum-catalyzed hydrosilylation, followed by vacuum impregnation, fully in situ gel infiltration, and curing inside the three-dimensional pore network of the high-porosity needle-punched quartz fiber felt. Subsequent stepwise heating to 1400 °C in high-purity argon accomplished pyrolysis and transformed the organic skeleton into an inorganic ceramic phase. The as-obtained lightweight integrated composite (optimized formulation designated LHRC-05) featured a highly porous, thermally insulating skeleton with balanced mechanical reinforcement, while the pure PCS sample was prepared as the control and coded as LHRC-0, The material preparation process is illustrated in Figure 6.

The room temperature mechanical properties of needle-punched quartz felt-reinforced aerogels are governed by the intrinsic characteristics of the aerogel matrix and the fiber–matrix interfacial bonding strength. Appropriate PHMS addition improved the mechanical performance of the aerogel composite at a comparable density relative to the PCS/V4 binary matrix (LHRC-0). The basic properties of the integrated composites are summarized in Table 4. Compared with LHRC-0, LHRC-05 showed only a slight density increase from 0.38 to 0.40 g cm^−3^, while its room temperature compressive strength was significantly enhanced from 1.90 to 7.97 MPa, and its compressive modulus was increased from 80 to 180 MPa. This outcome arose from the synergistic effects of a refined three-dimensional matrix network and strengthened fiber–matrix interfacial adhesion.

**Figure 7:**
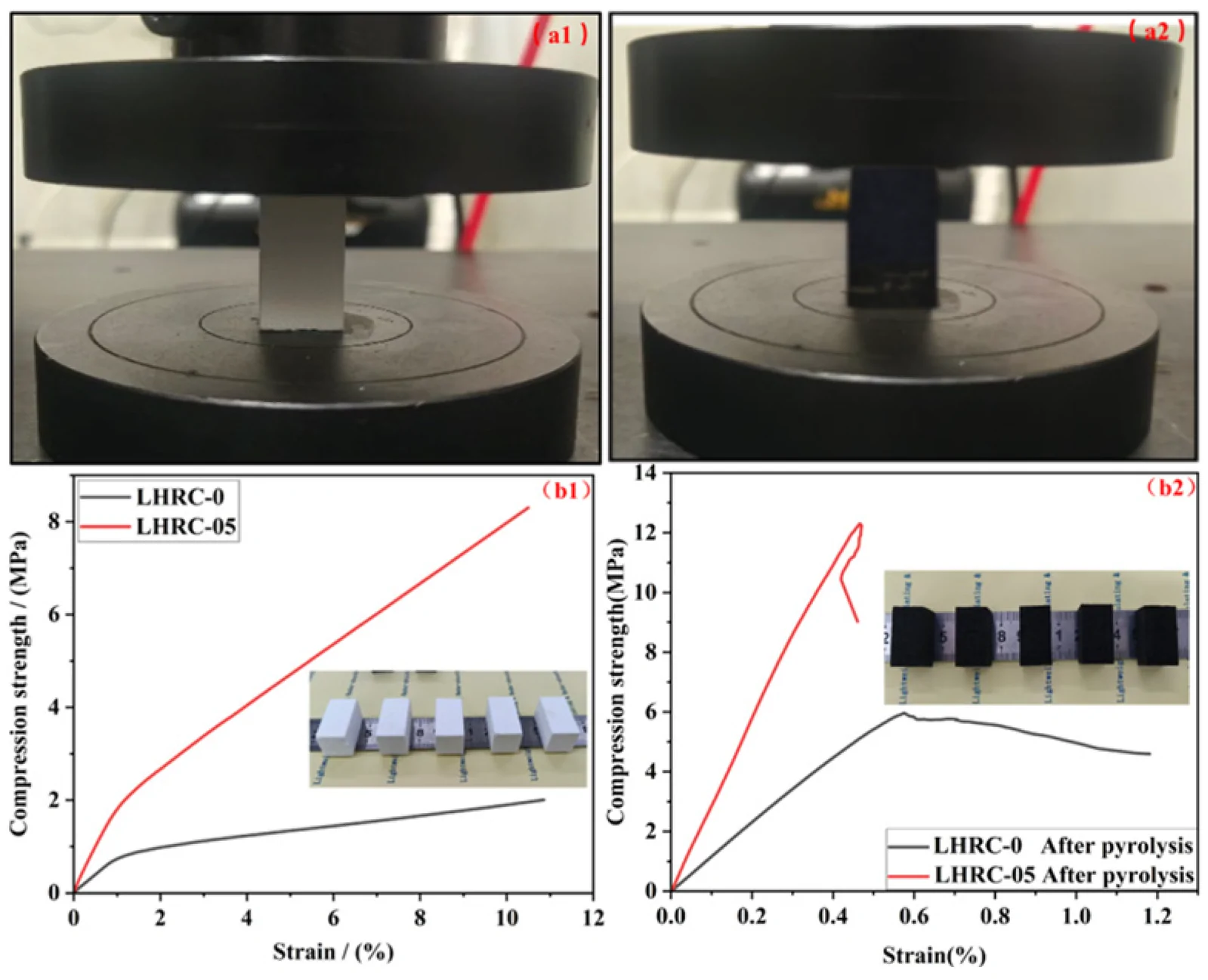
(**a1**,**a2**) Testing of mechanical properties. (**b1**,**b2**) Stress–strain curves of composite aerogels.

The pure PCS precursors formed rigid networks dominated by Si–C bonds after hydrosilylation crosslinking, with limited molecular chain mobility that generated local high crosslink agglomeration zones and low crosslink defect regions. As shown in Figure 7(b1,b2), microcracks rapidly propagated along these defects and triggered irreversible damage under external load. PHMS incorporation introduced flexible Si–O–Si chain segments to the PCS network through hydrosilylation, constructing hybrid crosslinked systems with balanced rigidity and flexibility. Conformational adjustment of the flexible segments accelerated molecular diffusion during crosslinking, markedly homogenizing the spatial distribution of crosslinking points and eliminating stress concentration defects. Meanwhile, the high bond-angle freedom of Si–O bonds endowed the matrix with moderate plastic deformation capacity, synchronously raising the intrinsic matrix strength and toughness.

From the fiber–matrix interface perspective, the introduction of PHMS markedly improved the infiltration and interfacial adhesion between the precursor and needle-punched quartz felt. Quartz fibers were terminated by surface silanol (Si–OH) groups, whereas neat PCS was a hydrophobic Si–C resin with poor polarity matching, leaving capillary voids within tows and needle-punched zones. The addition of PHMS—a polysiloxane bearing Si–H side groups—lowered the surface tension of the hybrid resin to 16–21 mN m^−1^, thereby improving wetting and enabling the sol to penetrate deeper into fiber bundles and micro-voids. More importantly, the Si–H functionalities of PHMS coupled with surface Si–OH groups on quartz filaments during curing and the early stage of pyrolysis, expected to form a chemically bonded Si–O–Si transition layer at the interface. Although direct interfacial spectroscopy was not performed, the markedly improved wetting (surface tension 16–21 mN m^−1^) and the enhanced interfacial adhesion are consistent with this proposed interfacial coupling mechanism. After hydrosilylation crosslinking and ceramization, this transition layer became a continuous graded SiC/SiOC interphase that bridged the fiber and the aerogel matrix. Loads were therefore efficiently transferred from the low-modulus aerogel skeleton to the high-strength quartz filaments across the interface, fully exploiting the toughening effect of continuous felt [3]. In contrast, the PCS/V4 binary matrix lacked such reactive coupling sites; its weak physical contact with fiber surfaces led to interfacial debonding and delamination under compression. As a consequence, the structural contribution of the fiber felt could not be mobilized.

For extreme high-temperature service scenarios, the retention of mechanical properties after high-temperature pyrolysis is a core index that determines engineering applicability [38,39]. Following pyrolysis at 1400 °C in high-purity argon, both groups completed the conversion of organic precursors into ceramic phases, with the compressive strength and modulus increasing markedly. The performance advantage of the PHMS-modified system was further amplified at high temperature: LHRC-05 reached a compressive strength of 12.3 MPa, which is 2.06 times that of LHRC-0 (5.96 MPa), and a compressive modulus of 2880 MPa, which is 2.46 times that of the pure system (1170 MPa). This result confirmed that the multiphase SiOC skeleton not only optimized room temperature performance but also markedly improved the structural stability and load-bearing capacity of the ceramicized framework at high temperatures.

### 3.4. Ablation Resistance, Thermal Insulation, and Heat Transfer Mechanisms of Fiber-Reinforced SiC/SiOC Aerogels

Having established the room temperature mechanical and interfacial benefits of the PHMS-tailored matrix, this section evaluates the high-temperature service behavior of LHRC-05, focusing on elevated temperature thermal conductivity, arc-jet ablation performance, and the underlying heat transfer and oxidation mechanisms.

**Figure 8:**
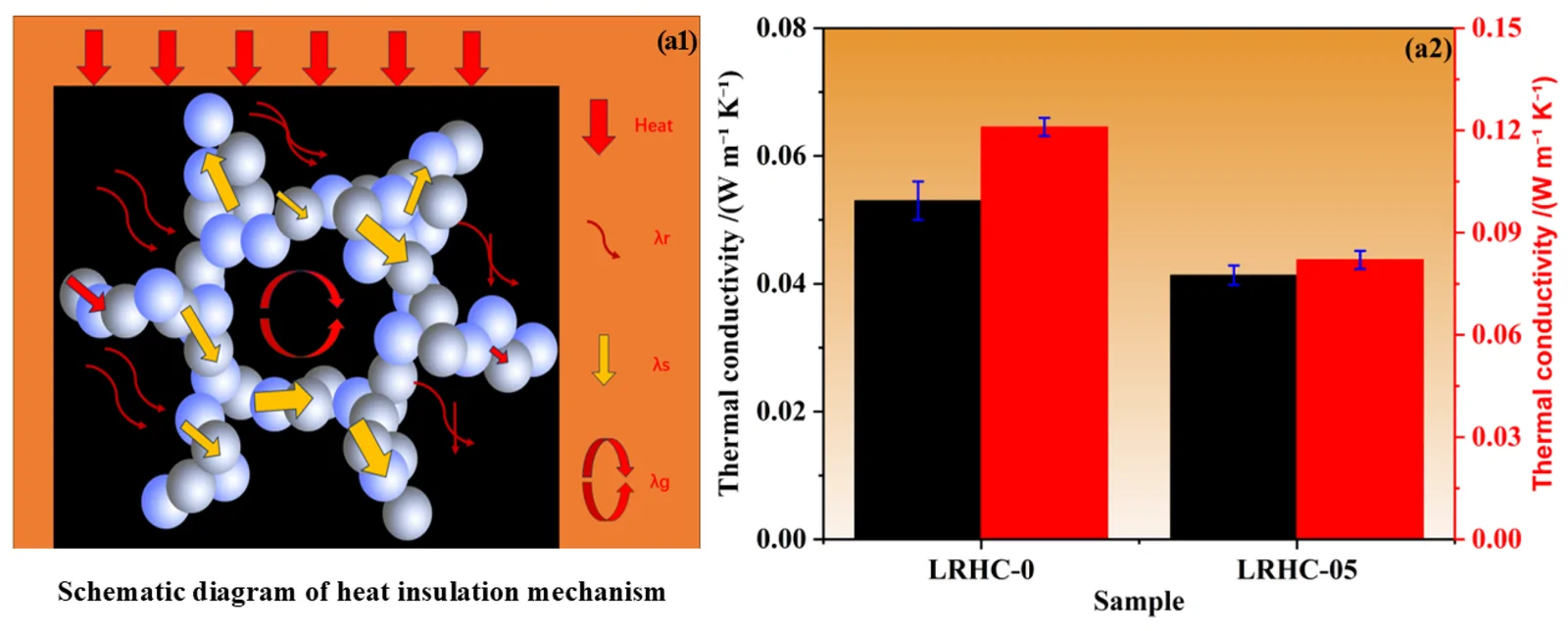
(**a1**) Heat transfer mechanism in aerogels. (**a2**) Steady-state thermal conductivity of samples at different temperatures.

The multiphase SiOC skeleton suppressed thermal conduction through synergistic attenuation of solid, gaseous, and radiative channels (Figure 8(a1)) [40,41]. The total effective thermal conductivity λ_t_ is the superposition of solid (λ_s_), gaseous (λg), and radiative (λᵣ) contributions, i.e., λ_t_ = λ_s_ + λg + λᵣ. Two comparative samples with identical high-porosity needle-punched quartz fiber felt were designed: LHRC-0 (PCS/V4 binary SiC aerogel) and LHRC-05 (PCS/PHMS/V4 ternary hybrid matrix). As shown in Figure 8(a2), LHRC-0 exhibited a room temperature thermal conductivity of 0.053 W m^−1^ K^−1^, which rose sharply to 0.121 W m^−1^ K^−1^ at 1000 °C. By contrast, LHRC-05 delivered a lower room temperature thermal conductivity of 0.042 W m^−1^ K^−1^, which increased only to 0.082 W m^−1^ K^−1^ at 1000 °C. This demonstrated that PHMS incorporation effectively suppressed the effective thermal conductivity of the hybrid composite at both room and elevated temperatures.

At 1000 °C, λᵣ superseded λg and λ_s_ as the dominant heat transfer channel. Thus, the high-temperature thermal conductivity is essentially governed by whether the nanoporous architecture can survive and continue to scatter thermal photons. For LHRC-0, pure PCS-derived SiC skeleton progressively sintered and coarsened upon prolonged exposure to 1000 °C: the ligaments densified, the interparticle necks thickened, and the pores grew toward sub-micrometer scales, sharply reducing the density of refractive index discontinuities that scattered mid- and far-infrared photons. The aerogel thus became semi-transparent to thermal radiation, and λᵣ—and hence the overall thermal conductivity—increased steeply with temperature. In contrast, the PHMS-optimized SiC/SiOC hybrid skeleton of LHRC-05 retained its refined nanoscale architecture up to 1000 °C. The SiOC-rich amorphous phase acted as a sintering barrier that suppressed the coarsening of SiC-rich domains and the thickening of interparticle necks. As such, the nanopore distribution and ligament size established at the optimum PHMS loading were largely preserved. The abundant heterogeneous boundaries between SiC-rich and SiOC-rich domains within the skeleton remained stable, providing persistent wavelength-matched scattering interfaces for infrared photons within pores. The synergistic retention of a narrow nanopore distribution, a nanoscale skeleton, and a high density of internal phase interfaces therefore simultaneously suppressed λg, λ_s_, and, most importantly, λᵣ at 1000 °C—an effect that accounted for the markedly lower high-temperature thermal conductivity of LHRC-05 relative to LHRC-0.

**Figure 9:**
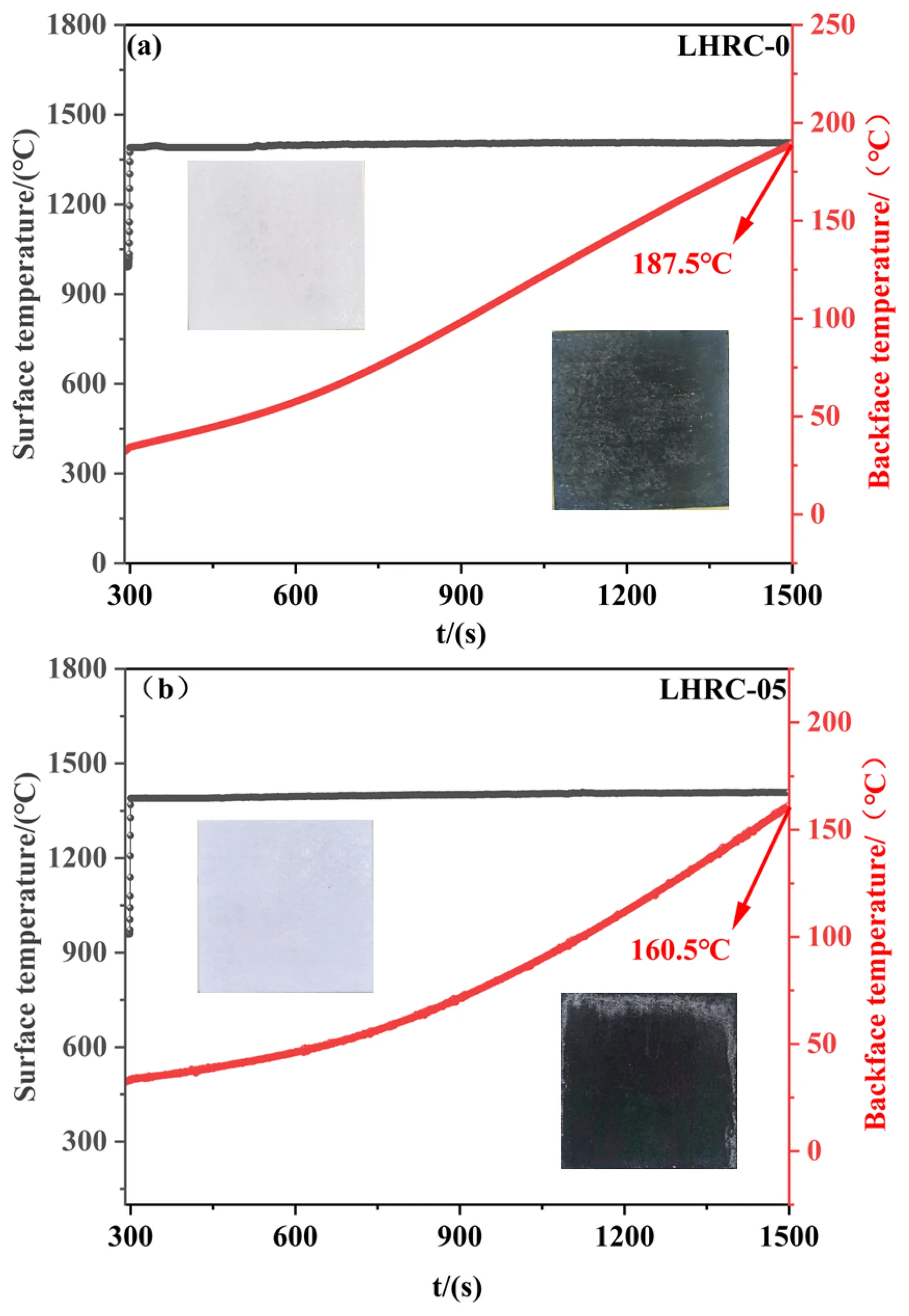
Curves of surface temperature and back-face temperature rise of LHRC samples. (**a**) LRHC-0; (**b**) LRHC-05.

The optimized LHRC-05 exhibited outstanding arc-jet ablation performance and long-duration thermal protection under aerobic conditions, following the protocol established for the arc-heated assessment of fiber-reinforced ceramic insulators [42]. Figure 9a presents digital photographs of the specimen before and after ablation, together with the front-surface and back-face temperature histories. Under a total heat load of 500 MJ m^−2^ accumulated over 1500 s, the surface temperature stabilized at approximately 1400 °C, while the maximum back-face temperature rise across a 25 mm thick specimen was only 160.5 °C. The linear ablation rate was as low as 0.53 μm s^−1^, accompanied by a mass ablation rate of merely 0.021 g s^−1^, corresponding to near-zero volumetric recession [43]. To place these values in context, the fiber-reinforced pure SiC control (LHRC-0) was tested under identical arc-jet conditions. Table 5 summarizes the exposed area, initial and final masses, thickness, linear and mass ablation rates, and back-face temperature rise for both LHRC-0 and LHRC-05, together with the measurement uncertainty.

**Figure 10:**
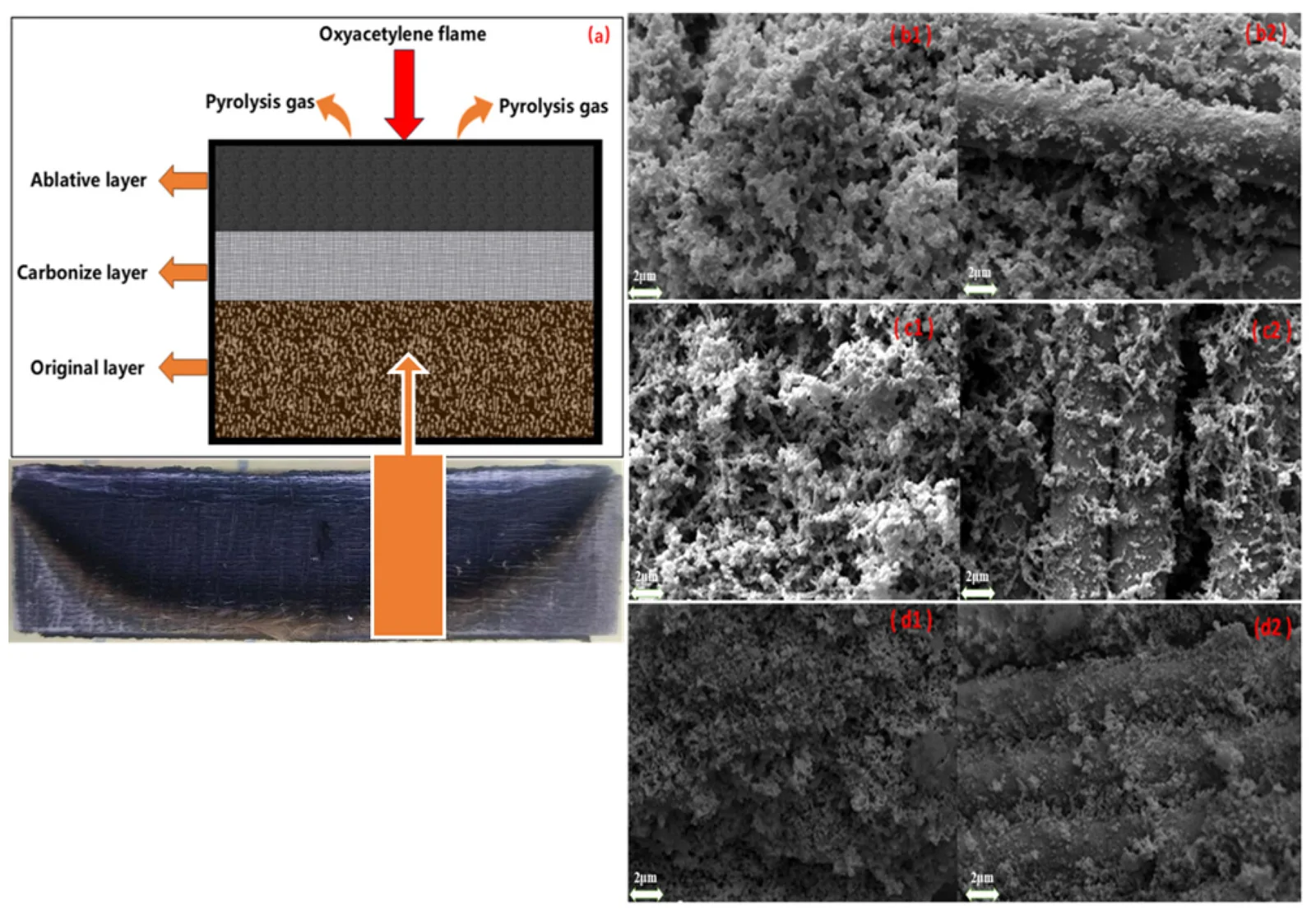
(**a**) Schematic diagram of ablation mechanism under arc-heated wind tunnel qualification test and ablated cross-sectional morphology. SEM images of different layers after ablation: (**b1**,**b2**) ablation layer; (**c1**,**c2**) transition layer; (**d1**,**d2**) virgin matrix layer.

Post-ablation inspection revealed that the macroscopic morphology remained intact, with the surface free of distinct ablation grooves, penetrating cracks, or large-area spalling. Cross-sectional analysis (Figure 10a–d) further disclosed a well-defined layered architecture comprising a dense ablative protective layer, a transitional carbonized layer, and an undamaged original porous substrate—morphology typical of PDC-based ablatives under plasma exposure [44]. The interlayer interfaces exhibited continuous transitions without cracking or delamination, and the porous skeleton architecture of the internal fiber mats was effectively preserved.

The superior ablation performance originated from the high-temperature oxidation and self-healing behavior of PHMS-derived SiC/SiOC hybrid skeleton. As a silicon–oxygen source in a ternary precursor system, PHMS participated in hydrosilylation crosslinking during sol–gel processing, embedding abundant Si–O bonds into the organic–inorganic hybrid network and thereby modifying the post-pyrolysis phase constitution and high-temperature response. Under the high-enthalpy arc-jet flow at 1400 °C, the outermost SiC-rich domains, together with carbonaceous species, were expected to undergo passive oxidation, in situ generating a dense adherent silica-rich glass scale [45,46]. Consistent with established observations on SiOC monolithic and plasma-exposed SiC, this oxide layer acted as an efficient barrier to inward oxygen diffusion, arresting further oxidation of the underlying skeleton and accounting for the extremely low linear ablation rate and near-zero volumetric recession [44,45]. Moreover, the viscous oxide products possessed favorable high-temperature fluidity and wettability that enabled them to fill microcracks and pore defects generated by thermal stress and aerodynamic erosion, thereby realizing a microscopic self-healing effect during ablation—analogous to the silica layer healing reported for long-life anti-oxidation systems [47]. The SiOC-rich amorphous phase, with its high softening temperature and moderate viscosity at 1400 °C, sintered and densified the oxidized surface layer rather than allowing it to spall under the combined thermal and aerodynamic shear loads [44,45]. The quartz fiber reinforcement further dissipated the through-thickness thermal gradient and alleviated thermal mismatch stresses, suppressing the formation of radial grooves and surface cracks during rapid heating and cooling transients [48,49]. The synergistic combination of a self-healing oxide scale, a thermally stable glassy matrix, and a stress-relieving fiber architecture, therefore, endowed LHRC-05 with excellent ablation resistance and thermal insulation for long-duration hypersonic service.

## 4. Conclusions

In this work, lightweight, heat-insulating, and ablation-resistant composites (LHRCs) were fabricated using a ternary PCS/PHMS/V4 precursor system infiltrated into high-porosity needle-punched quartz fiber felts. The role of PHMS content in governing the microstructural evolution and macroscopic properties of SiC/SiOC hybrid aerogels was systematically investigated. The main findings are presented below:

(1) PHMS acted as a phase and microstructure regulator, forming a molecularly interpenetrated three-dimensional network through hydrosilylation between Si–H and vinyl groups. Increasing PHMS loading raised the crosslinking density, suppressed capillary-induced collapse during ambient pressure drying and mitigated pyrolytic shrinkage. At the optimal PCS/PHMS mass ratio of 1:0.5 (SPA050), the pyrolysis shrinkage was reduced to 20% and a well-developed nanoporous architecture was retained.
(2) The optimized ceramic SiC/SiOC aerogel (SPA050) exhibited a room temperature compressive strength of 24.20 MPa, 128.7% higher than that of the pure SiC counterpart (SPA0, 10.58 MPa), together with a low room temperature thermal conductivity of 0.035 W m^−1^ K^−1^ that only rose to 0.082 W m^−1^ K^−1^ at 1000 °C. (We note that the value of 7.83 MPa in Table 2 refers to the precursor organic aerogel SP050, whereas Table 2 reports the ceramic SPA050 value of 24.20 MPa.) The enhanced insulation is attributed to phonon scattering at the multiphase SiC/SiOC interfaces and suppressed gaseous conduction within the nanopores.
(3) The fiber-reinforced LHRC-05 composite combined a low bulk density of 0.40 g cm^−3^ with a through-thickness compressive strength of 7.97 MPa, which further increased to 12.3 MPa after pyrolysis at 1400 °C. In ground-based arc-jet tests (500 MJ m^−2^, 1500 s, ~1400 °C surface), a 25 mm thick LHRC-05 panel showed a back-face temperature rise of only 160.5 °C, a linear ablation rate of 0.53 μm s^−1^, and a mass ablation rate of merely 0.021 g s^−1^, corresponding to near-zero volumetric recession. The Si–O species introduced by PHMS were expected to promote the in situ formation of a viscous silica-rich oxide layer at elevated temperatures, which filled microcracks and thereby improved high-temperature oxidation and ablation resistance.
(4) At the fiber–matrix interface, PHMS lowered the surface tension of the hybrid resin to the 16–21 mN m^−1^ range, improving wetting and sol penetration into quartz fiber bundles; meanwhile, the Si–H functionalities of PHMS are proposed to couple with surface Si–OH groups on quartz filaments, potentially forming a chemically bonded Si–O–Si interphase (an interpretation supported by the observed improvement in wetting and interfacial adhesion, although direct interfacial spectroscopy was not performed) that bridges the fiber and aerogel matrix and enables efficient load transfer. These results demonstrated that the PHMS-tailored SiC/SiOC hybrid aerogel composite addresses the key limitations of conventional thermal protection materials—functional singularity, high-temperature degradation of insulation and ablation resistance, and weak fiber–matrix bonding—offering a feasible strategy for multifunctional integrated thermal protection in aerospace and other extreme high-temperature applications.

## Figures and Tables

**Table 1:** Consolidated testing conditions.

Characterization Tests	Method/Standard	Specimen Dimensions	Test Conditions
Density	GB/T 1463-2005	100 × 50 × 50 (mm)	Rt, air
Compressive properties	GB/T 1448-2005	15 × 15 × 30 (mm)	Rt, air
Thermal conductivity (RT)	ISO 22007-2 (Hot Disk TPS2500S)	100 × 100 × 7 (mm)	Rt, air
Thermal conductivity (high-T)	ISO 22007-2 (Hot Disk TPS2500S)	100 × 100 × 7 (mm)	1000 °C, Ar

**Table 2:** The performance data for hybrid aerogels.

Sample	Volume Density (g/cm^3^)	Compression Strength (MPa)	Compression Modulus (MPa)	Thermal Conductivity (W/m·K)
SP0	0.18	2.12	20	0.032
SP025	0.19	2.72	40	0.035
SP050	0.21	7.83	70	0.032
SP075	0.20	6.45	65	0.034
SP1	0.20	3.98	45	0.035

**Table 3:** The performance data of hybrid aerogels after pyrolysis.

Sample	Volume Density (g/cm^3^)	Compression Strength (MPa)	Compression Modulus (MPa)	Thermal Conductivity (W/m·K)	Shrinkage Ratio (%)
SPA0	0.25	10.58	140	0.045	28
SPA025	0.26	16.55	190	0.038	23
SPA050	0.24	24.20	250	0.035	20
SPA075	0.27	19.62	360	0.039	26
SPA1	0.28	14.34	140	0.050	26

**Table 4:** Basic performance data of integrated materials.

Sample	Volume Density (g/cm^3^)	Compression Strength (MPa)	Compression Modulus (MPa)	Compression Strength (MPa)	Compression Modulus (MPa)
LHRC-0	0.38	1.90 (RT)	80 (RT)	5.96 (1400 °C)	1170 (1400 °C)
LHRC-05	0.40	7.97 (RT)	180 (RT)	12.3 (1400 °C)	2880 (1400 °C)

**Table 5:** Arc-jet ablation comparison of LHRC-0 and LHRC-05.

Parameter	LHRC-0	LHRC-05	Uncertainty
Exposed area (mm^2^)	100 × 100	100 × 100	±5%
Thickness (mm)	25	25	±0.1
Initial mass (g)	98.34	101.26	±0.1
Final mass (g)	60.09	69.76	±0.1
Linear ablation rate (μm s^−1^)	0.8	0.53	±0.1
Mass ablation rate (g s^−1^)	0.0255	0.0210	±0.01
Max. back-face ΔT (°C)	187.5	160.5	±1

## Data Availability

The original contributions presented in this study are included in the article. Further inquiries can be directed to the corresponding authors.
